# The Attentional Window Modulates Capture by Audiovisual Events

**DOI:** 10.1371/journal.pone.0039137

**Published:** 2012-07-10

**Authors:** Erik Van der Burg, Christian N. L. Olivers, Jan Theeuwes

**Affiliations:** Department Cognitive Psychology, Vrije Universiteit, Amsterdam, The Netherlands; University of California, Davis, United States of America

## Abstract

Visual search is markedly improved when a target color change is synchronized with a spatially non-informative auditory signal. This “pip and pop” effect is an automatic process as even a distractor captures attention when accompanied by a tone. Previous studies investigating visual attention have indicated that automatic capture is susceptible to the size of the attentional window. The present study investigated whether the pip and pop effect is modulated by the extent to which participants divide their attention across the visual field We show that participants were better in detecting a synchronized audiovisual event when they divided their attention across the visual field relative to a condition in which they focused their attention. We argue that audiovisual capture is reduced under focused conditions relative to distributed settings.

## Introduction

Every day we receive a bulk of information from different sensory modalities. It has been extensively demonstrated that these sensory inputs interact when presented in either close temporal or spatial proximity (see e.g. [1], [2], for recent reviews). So far, most studies investigating multisensory interactions have used sparse displays, often involving a single visual event in combination with a single auditory event at a time (see e.g. [3], [4]. Whereas these studies were successful in reporting multisensory interactions, they say little about how we behave and integrate information from different sensory modalities in a competing environment (see also [5], for this argument).

Recently we have shown that auditory and tactile signals can affect the competition among multiple visual events in dynamic environments [6], [7]. In the Van der Burg et al. [6] study, participants searched for a horizontal or vertical line segment among up to 48 other distractor line segments of various orientations, all continuously changing color. We found that both search time and search slopes were drastically reduced when the target color change was accompanied by an auditory signal compared to a condition in which no such signal was present (see also [8]). This audition driven visual search benefit, which we called the “pip and pop” effect, was observed even though the auditory signal was uninformative about the orientation, color and, most important, the location of the synchronized visual target. One might argue that the auditory signal acted as a temporal cue. The tone may have informed participants about when the target changed color, so that it became easier to find. However, in follow-up experiments we found evidence that the search benefits were not due to temporal cueing. For instance, we presented similar temporal information by briefly making the fixation dot disappear or by briefly presenting a peripheral halo when that visual target changed color. In both cases, the temporal cue did not affect visual search performance at all, while, in a control experiment, we showed that these temporal visual cues were effective temporal warning signals (see also Van der Burg et al. 2008b for a detailed discussion about temporal cueing).

In a subsequent study, we measured event-related brain potentials (ERPs) to investigate the underlying neural mechanism of the pip and pop effect [9]. In the Van der Burg et al. (2011) study, we reported that the search benefits correlated with an early (50–60 ms) multisensory interaction over the left parieto-occipital cortex. This early multisensory interaction was then followed by an early (80–100 ms) modulation over the occipital areas contralateral to the synchronized visual target, and an enhanced N2pc (∼200 ms), indicating that the synchronized visual event was affected by the auditory signal and as a result captured attention, respectively. Interestingly, a similar early multisensory interaction as well as a reliable modulation over the occipital cortex and N2pc were observed in the case that a task irrelevant distractor change was accompanied by an auditory signal, suggesting that the pip and pop effect occurred in a stimulus-driven, automatic fashion (see also [6], [10]). The presence of an early multisensory interaction in the case that a single distractor was synchronized with an auditory signal bolsters the claim that the pip and pop effect is not due to temporal cueing, since the tone was never synchronized with the target in these distractor blocks, and participants were aware of this. In other words, there was no need for participants to attend to the auditory signal, and to use the onset of the auditory signal as a time marker.

Even though the above results provide behavioral as well as neurophysiological evidence that synchronized audiovisual events capture attention in an automatic manner (see also [11]), some results suggest that the capture by such events is not as strong as previously reported attentional capture effects within purely the visual domain (e.g. for color [12] or for abrupt onset [13]). For instance, search slopes for targets in the synchronized conditions never approached zero (see e.g [6], [7]), as would have been indicative of complete prioritization. Within the visual domain, Belopolsky and colleagues [14], [15] recently provided evidence that the extent to which attention is divided across the visual field (referred to as the attentional window) modulates the degree to which salient events capture attention (see also [16]). In these studies Belopolsky and colleagues provided evidence for the idea proposed by Theeuwes ([17], p. 436) arguing that *“top-down control over visual selection can be accomplished by endogenously varying the spatial attentional window”* (see also [18], [19]). Specifically, Belopolsky et al. [14] showed that salient singletons capture attention when participants adopt a large, diffuse attentional window (i.e. when participants attend to the whole display), while the same singletons do not capture attention when participants adopt a small, more focused attentional window (i.e. when participants attend to the center of the screen only) (see also [20]–[22]). It is possible that the size of the visual attentional window also plays a role in the pip and pop effect. For example, if, on some trials, observers adopt a relatively small attentional window (e.g. due to a distractor color change that captures attention), they may miss the visual target event (if it is located outside their attentional window), generating relatively long search times on some trials (since participants must wait for the subsequent audiovisual event to occur). The present study was designed to investigate whether the size of the attentional window has an effect on the extent to which audiovisual events capture attention.

## Experiment 1

**Figure 1:**
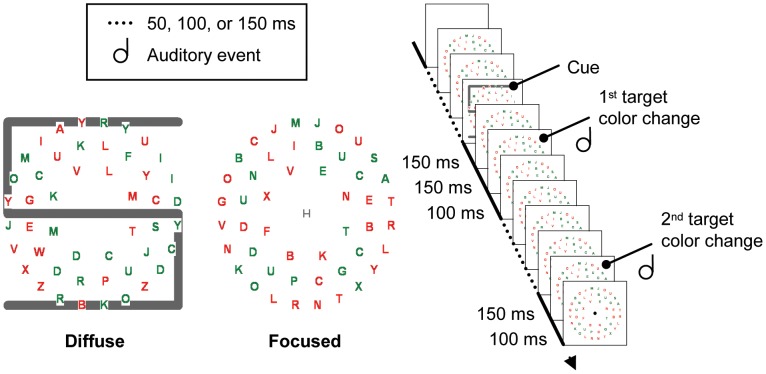
Example of the search display used in the present study. Participants were asked to do a letter matching task determining whether the target letter (i.e. the letter S or H) was the same or different letter as the cue letter, which was also an S or H.In the diffuse attentional window condition, this cue was a large letter. In the focused attentional window condition, this cue was a small letter.

Participants searched for a target letter S or H among distractor letters. Importantly, before searching, participants were asked to memorize a letter S or H (the cue), which was necessary to make a correct response. Participants were asked whether the target in the search display was the same letter as the cue or a different letter. The important manipulation involved the size of the cue. In the diffuse attentional window condition, the cue was a large letter, subtending the entire area behind the search display. In the focused attentional window condition, the cue was a small letter presented at the center of the visual display. Figure 1 presents an example of the visual search display used in the present study.

Irrelevant to the task, at random intervals a random number of distractor letters changed color (from green to red or vice versa). On average once every 900 ms, the target letter changed color, and it did so alone. This unique target color change was always accompanied by an auditory signal. We expected that this synchronized audiovisual event would capture attention [6]. Importantly, if the size of an attentional window modulates capture by audiovisual events, then we expect better performance when subjects adopt a distributed, more diffuse attentional window than when participants adopt a small, focused attentional window. Moreover, the letter cue was only presented once (300 ms prior to the first target color change, with a duration of 150 ms). Therefore, if setting the attentional window is only effective during a particular time window, one expects that this manipulation will affect capture immediately following the attentional window manipulation (i.e., the first synchronized audiovisual event) but not so much for audiovisual events later in time.

**Figure 2:**
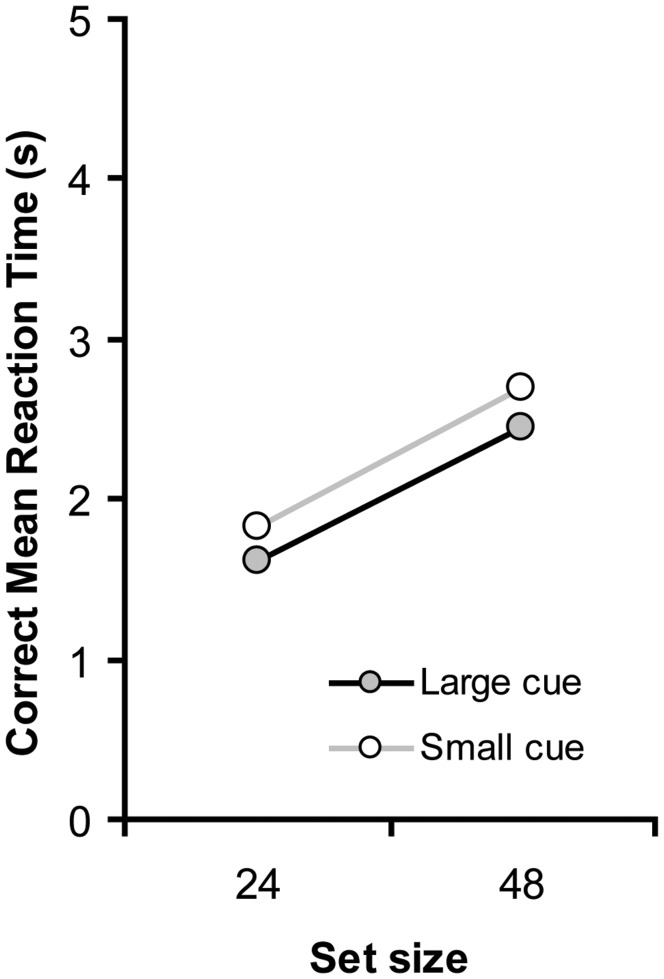
Correct mean reaction time (RT) as a function of a focused mode (small cue) or a diffuse mode (large cue). Note that the RT reflects the time to respond to the visual target from the presentation of first target color change.

### Methods

#### Ethics statement

Written consent was obtained from each participant prior to the experiments. The experiments were approved by the local ethics committee of the Vrije Universiteit.

#### Participants

In Experiment 1, eleven participants (6 female; mean age = 22.3; ranging from 19–27 years) participated. One participant was excluded from further analysis because of an overall high error rate (>15%). In Experiment 2, ten new participants (5 female; mean age = 20.7; ranging from 18–24 years) participated. Participants were paid €7 an hour.

#### Stimuli and apparatus

Experiments were run in a dimly lit, air-conditioned cabin. Participants were seated approximately 80 cm from the monitor and wore Sennheiser HD 202 headphones. The auditory stimulus was a 500 Hz tone (44.1 kHz sample rate; 16 bit; mono) with a duration of 60 ms (including a 5 ms fade-in and fade-out to avoid clicks) presented on the headphones. The visual search displays consisted of 24 or 48 red (13.9 cd m^−2^) or green (46.4 cd m^−2^) capital letters (Font type: “Arial”; height 0.7°; width 0.6°) from the alphabet on a black (<0.05 cd m^−2^) background. Color of each letter was randomly determined. All letters were placed on three imaginary circles with a radius of 3.0°, 4.6°, and 6.2° centered around the center of the display, with the constraint that the number of letters for each imaginary circle was limited to 8, 16 and 24 letters, respectively. The distractor letters were randomly determined letters from the alphabet, except the letters S and H which were used as target letters. The display changed continuously at randomly determined intervals of 50, 100, or 150 ms, with the constraint that the target color change was preceded by a 150 ms interval and followed by a 100 ms interval. At the start of each interval, a randomly determined number of distractor letters changed color (from green to red or vice versa), within the following constraint: When set size was 24, the number of letters that changed was 1, 2, or 3. When set size was 48, the number of letters that changed was 1, 4, or 7. Note that the target was not the only visual event that changed alone. Furthermore, the target letter always changed alone and could change on average once every 900 ms (1.1 Hz; minimum = 500 ms; maximum = 1,300 ms). In Experiment 1, the target color change was always accompanied by the auditory signal, and participants were informed about this. In Experiment 2, the tone was always absent. The target letter could not change during the first 5 display changes (on average 500 ms). Note that participants were able to do the task without any color changes (e.g. prior the first target color change) since the target was always present. On each trial, the cue, which was another letter S or H was presented. The first target color change was always preceded by two 150 ms intervals. The cue was presented during the first 150 ms interval (which is equivalent to 300 ms before the target color change). The cue was briefly presented (for 150 ms) to make sure that participants immediately processed the cue. The cue was light grey either small (width 0.4°; height 0.4°) or large (width 13.0°; height 13.0°) and presented at the center of the display for a fixed duration of 150 ms.

#### Design and procedure

The set size was either 24 or 48. The other manipulation involved the size of the cue (small or large). Participants were asked to memorize the cue, as this was necessarily to make a correct response to the target letter. Dependent variables were the reaction time (RT) and accuracy. Note that the RT reflects the time to respond from the first target letter color change. Each trial began with the presentation of a fixation dot for 500 ms at the center of the screen, followed by a 500 ms blank screen. Subsequently, the search display appeared until participants responded. Participants were instructed to press the *z*-key when the target letter was identical to the cue, or to press the/−key when it was not identical to the cue. The S and H were balanced for cue and target letter and randomly mixed within blocks of 24 trials each. In Experiment 1, participants received eight large-cue blocks and eight small-cue blocks in counterbalanced, alternating order, preceded by two practice blocks. In Experiment 2, participants received four large cue blocks and four small cue blocks in a counterbalanced, alternating order, preceded by two practice blocks. Participants were informed about the size of the cue prior to each block, and received feedback about their overall mean accuracy and overall RT after each block.

### Results and Discussion

The RT results are presented in Figure 2. RT data from practice blocks and erroneous trials were excluded. RTs and Errors were subjected to a repeated measures univariate analysis of variance (ANOVA) with set size (24 vs. 48) and cue size (small vs. large) as within-subjects variables.

#### RT data

On average, RTs increased with increasing set size, *F*(1, 9) = 22.2, *p* = .001. Importantly, RTs were faster when the cue was large (2,035 ms) than when the cue was small (2,262 ms), *F*(1, 9) = 9.3, *p* = .01. The interaction between cue size and set size was not reliable (*F*<1).

#### Error data

Overall mean error rate was 6.4%. The main effect of set size was not reliable, *F*(1, 9) = 3.1, *p* = .110. Participants made less errors when the cue was large (5.3%) than when the cue was small (6.6%), *F*(1, 9) = 9.3, *p* = .01. The interaction was not reliable (*F*<1).

Responses to audiovisual events were faster and more accurate when attention was diffuse (large cue) than when attention was focused (small cue) at the center of the screen (as induced by the size of the cue). This is consistent with our notion that the size of the visual attentional window affects audiovisual integration. However, an alternative explanation for the better performance when attention is diffusely spread across the visual field is that it may be easier to process a large letter than a small letter. In a control experiment we tested whether a large cue may be processed more quickly than a small cue. To control for this alternative explanation, we replicated the experiment, except that participants (N = 8; 5 female; mean age 25.1 years; range 18–34 years) were asked to respond as fast as possible to the identity (S or H) of the cue, by pressing the corresponding (S or H) key. Furthermore, the tone was always absent. There was no significant cue size effect on RTs and Errors, *t*(7) = 1.6, *p* = .151, and *t*(7)<.624, *p* = .553, respectively. If anything, performance was better when the cue was small (496 ms; 3.7% errors) than when the cue was large (509 ms; 4.7% errors). We conclude that participants were not able to process a large cue any faster than a small cue, suggesting that differences in processing speed between the letters of different sizes cannot account for our findings.

Whereas Belopolsky et al. [14] demonstrated a Cue size × Set size interaction, the present study revealed a clear effect of cue size, but this effect was independent of the number of elements in the display. An important difference is that Belopolsky et al. used color singletons, which were always present, and therefore constantly salient. In contrast, capture by audiovisual events, as investigated here, has a discrete, “all or nothing” nature, because the visual target only becomes temporarily salient, at the moment of the color change, when accompanied by an auditory signal. If participants miss this temporally salient visual event (e.g. due to the size of the attentional window), they may wait for the second opportunity (the subsequent audiovisual event; on average 900 ms later) to detect the pop out, resulting in additive cue size and set size effects. One might argue that there was no need for the participants to wait for the second opportunity since the target was always present, also before any sound or color change occurred. However, given its serial nature, such a purely visual search would not result in an additive effect of the cue with set size. It appears that observers often preferred to wait for the next sound rather than engage in an effortful search.

**Figure 3:**
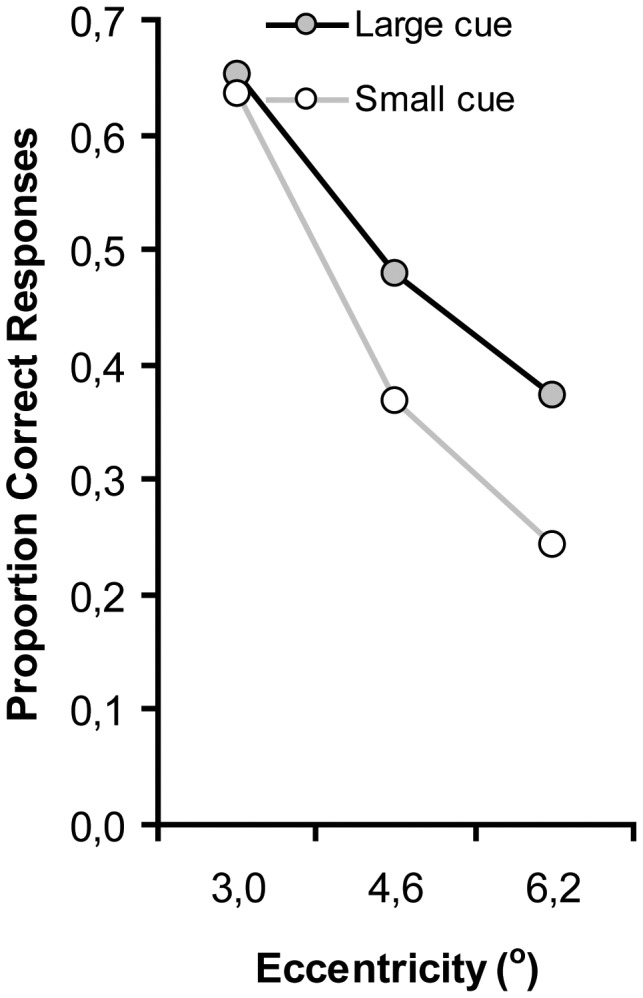
Proportion of correct responses faster than 1,200 ms after the first audiovisual event, as a function of cue size and eccentricity (distance between fixation and target location).

Further support for an effect of attentional window size on audiovisual attentional capture comes from an analysis of eccentricity effects. Eccentricity effects are expected to be reduced when attention is more diffuse (as induced by a large cue), whereas if attention is focused at central fixation (as induced by a small cue), especially targets at eccentricity should suffer [23]. We limited this analysis to only those responses that were made to the very first audiovisual target event (i.e. within 1200 ms post onset; note that target events occurred every 900 ms, but we assume that no reasonable response could occur within 300 ms). Because our cue was only brief (150 ms), we expected the attentional window manipulation to be only temporarily effective, but at the very least affect the first synchronized event. After this event, participants may have been able to return to their ‘default’ attentional window. Figure 3 presents the results (collapsed over set size). Proportion of responses were subjected to an ANOVA with cue size (small vs. large), and eccentricity (3.0°, 4.6° vs. 6.2°) as within-subjects variables.

Consistent with Carrasco et al. (1995), there was a reliable eccentricity effect as the proportion responses decreased with increasing eccentricity, F(2, 18) = 95.8, p<.001. The main effect of cue size was not significant, F(1, 9) = 3.3, p = .104. More importantly however, the interaction between cue size and eccentricity was reliable, *F*(2, 18) = 5.4, *p* = .01, confirming the notion that the size of the cue affected the attentional window (cue size) adopted by the participants. As is clear from Figure 3, the size of the attentional window affects capture by audiovisual synchrony, but only for targets presented far from fixation.

One potential caveat in the present experiment is that the result we obtained has nothing to do with the occurrence of the synchronized audio-visual event (the pip and pop effect) but would also occur in conditions in which no auditory signal is present. In other words, it is feasible that in conditions in which the target has to be detected by effortful serial search the window manipulation would generate a benefit for the diffuse relative to the focused condition. Experiment 2 was designed to rule out this possibility.

## Experiment 2

The present experiment was identical to the previous experiment, except that no tone was present. We expect slower search times and steeper search slopes relative to those obtained in Experiment 1, because the absence of the sound would require serial effortful search [6], [7]. If the attentional window manipulation is tied to the occurrence of the synchronized audio-visual event we expect that in this experiment in which no sound is presented, this manipulation should have no effect.

### Results and Discussion

**Figure 4:**
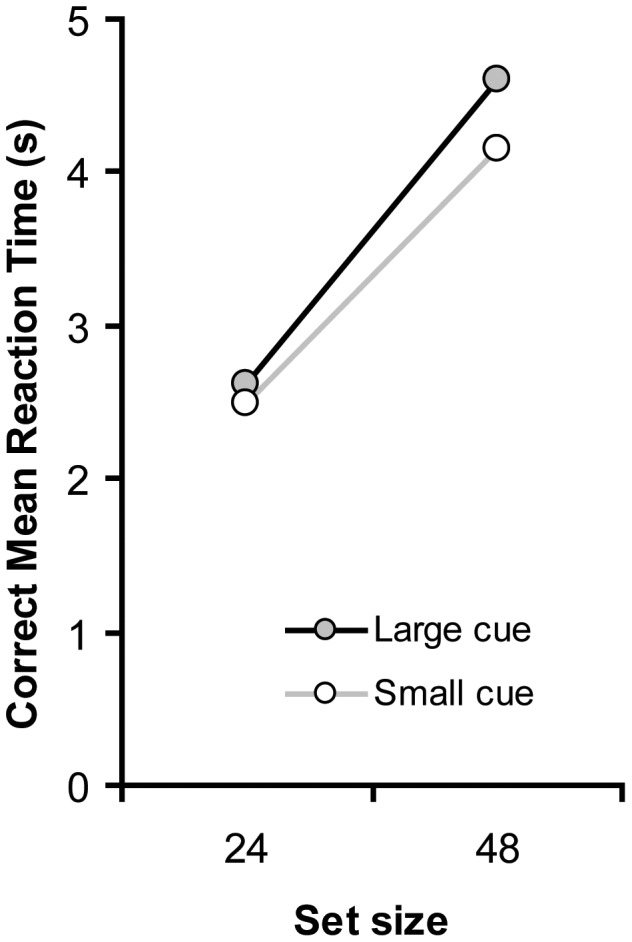
Correct mean reaction time (RT) as a function of set size and cue size.

The RT results are presented in Figure 4.

#### RT data

RTs increased with increasing set size, *F*(1, 9) = 37.7, *p*<.001. Contrary to Experiment 1, responses were slower when the cue was large (3,612 ms) than when the cue was small (3,318 ms). More importantly, the effect of cue size was not reliable, *F*(1, 9) = 2.4, *p* = .158. The interaction between cue size and set size was also not reliable (*F*<1).

#### Error data

Overall mean error rate was 4.8%. The ANOVA on Errors revealed no reliable main effect of set size, and cue size, *F*(1, 9) = 2.3, *p* = .163, and *F*(1, 9) = 1.4, *p* = .260, respectively. Neither was the interaction reliable (*F*<1).

As is clear from the RT and Error data, the cue size effect in Experiment 1 cannot be explained in terms of a purely visual mechanism of the attentional window on target detection, as there was no cue size effect in the present experiment (if anything the effect was in the opposite direction). Alternatively, one might argue that in Experiment 1 performance was better in the large cue condition because eyes tend to move when attention is distributed. In this case, RTs were faster in the diffuse attentional window condition since overt attention facilitated target detection. However, if the large cue was somehow beneficial in relation to eye movements, one would expect a similar benefit in Experiment 2, which was not the case.

**Figure 5:**
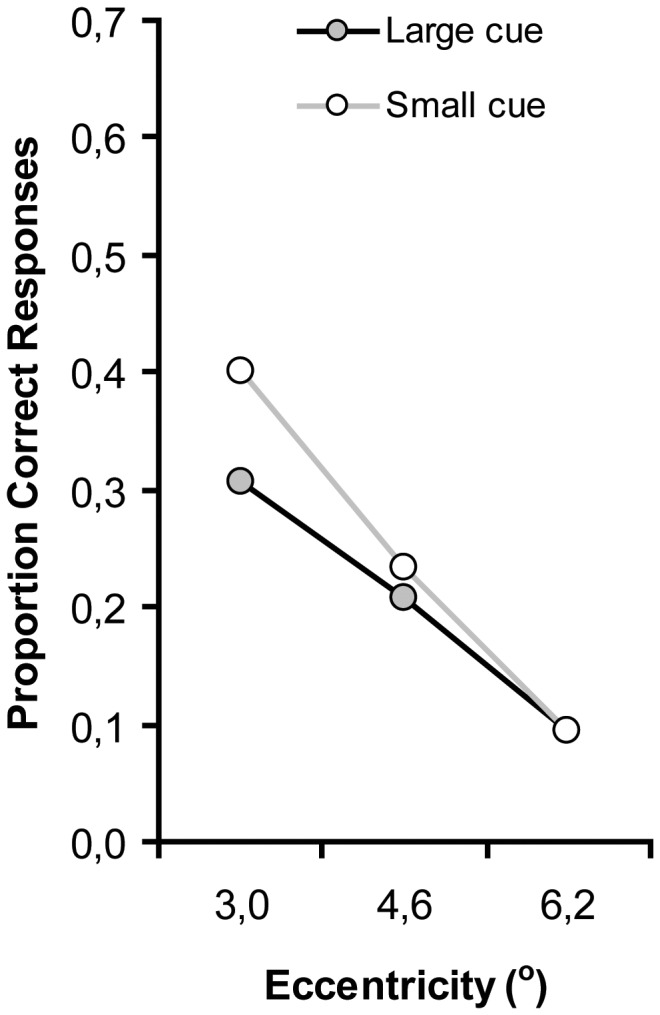
Proportion of correct responses faster than 1,200 ms after the first audiovisual event, as a function of cue size and eccentricity (distance between fixation and target location).

One might state that the actual attentional window effects were not observed, because the relatively small RT effect (of about 250 ms in Experiment 1) may have been obscured by the relative large search times in Experiment 2. Therefore, we conducted the same analysis as in Experiment 1 for the proportion of responses in which participants responded faster than 1,200 ms. Figure 5 presents the proportion of responses as a function cue size, and eccentricity (collapsed over set size).

There was a reliable eccentricity effect, F(2, 18) = 20.1, p<.001, as the proportion responses decreased as the target appeared further from fixation. There was no effect of cue size, F(1, 9) = 3.7, p = .085. Neither was there a significant Cue size × Eccentricity interaction, F(2, 18) = 2.2, p = .140. As is clear from Figure 5, the cue size had no temporal effect on the presentation of the visual target color change. If anything, the results suggest that participants were better when they were focused than when they were in a diffuse attentional mode, but only when the target appeared close to fixation. If one assumes that search requires serial effortful search (when no tone is present) then it is to be expected that when the focus is narrow people are faster (because one needs to search with a narrow focus) then when it is diffuse (people need to switch from diffuse to focus in order to find the target).

A between-experiment analysis was conducted with set size and cue size as within subjects variables. These analyses yielded a reliable effect of experiment, as RTs were faster when the target color change was accompanied by an auditory signal (2,148 ms; Experiment 1) than when no such signal was present (3,465 ms; Experiment 2), *F*(1, 18) = 9.2, *p*<.01. Furthermore, search slopes were overall shallower in Experiment 1 (35 ms per item) than in Experiment 2 (76 ms per item), as confirmed by a significant Experiment × Set size interaction, *F*(1, 18) = 7.7, *p*<.01. Thus, search was improved when a visual target was accompanied by a spatially uninformative auditory signal compared to the condition in which no auditory signal was present [6], [24]. Important, we observed a reliable Cue size × Experiment interaction, *F*(1, 18) = 6.5, *p*<.05, indicating a cue size effect in Experiment 1 (i.e. the size of the cue affects capture by audiovisual events), and no cue size effect in Experiment 2.

### Effect of Time

**Figure 6:**
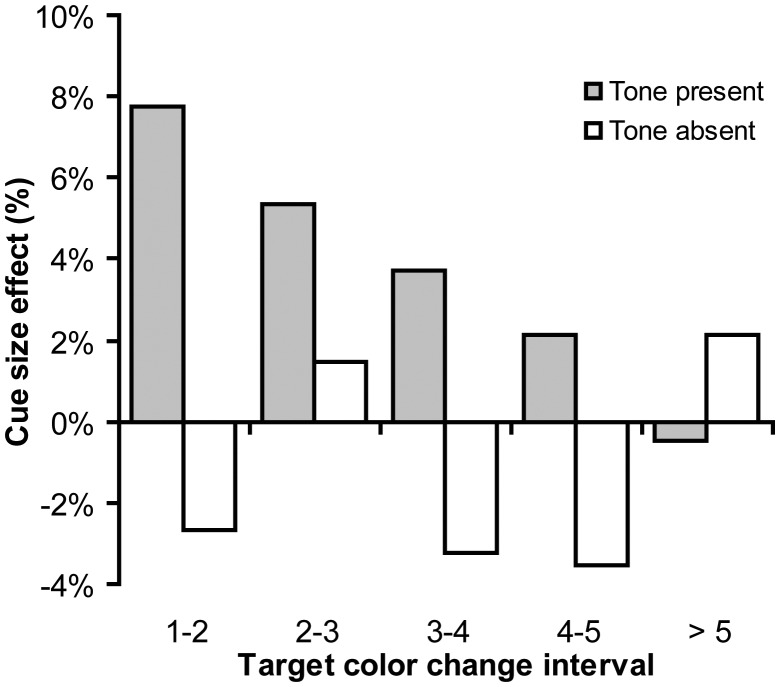
The effect of the size of the cue as a function of time (the interval in which the target changed), for the tone present and absent conditions. Cue size effect is the probability of correct response in the large cue condition – probability of correct response in the small cue condition. The data were pooled over set size.

It could well be that the cue size manipulation only had a temporary effect on the guidance by audiovisual integration. For example, a wide cue may initially induce a wide attentional window, but when observers subsequently revert to what is in essence a rather effortful visual search, the attentional focus may narrow down again. To make sure that participants processed the cue immediately, it was presented only briefly (for 150 ms) prior to the first target color change. Thus, the attentional window manipulation may have influenced mainly the first audiovisual events, after which participants returned to their default attentional window. Indeed, Fig. 6 suggests that this was the case. Figure 6 presents the probability of a correct response per target change interval, as a function of cue size, and as a function of tone presence. As is clear from Fig. 6, in the tone present condition, large cues were most effective between the first and the second audiovisual event and gradually decreased with time, indicating that the cue had only a temporary effect. This was statistically confirmed by two-tailed t-tests for each target change interval. The t-tests yielded a reliable cue size effect for the first interval (*t*(9) = 2.3, *p*<.05), but not for the remaining intervals (all *ts*<1.6, *ps* >.1). In contrast, in the tone absent condition, there was no cue effect (all *ts*<1.9, *ps* >.09).

## General Discussion

The present study replicated the pip and pop effect [6], [8], [10], [25], [26]. Search times as well as search slopes were markedly reduced when the target color change was accompanied by an auditory signal (Experiment 1) relative to a condition in which no such signal was present (Experiment 2). Important for the present question is that in the tone present condition, responses toward audiovisual events were overall faster when attention was distributed than when attention was focused (as induced by cue size). This effect cannot be explained in terms of a purely visual mechanism, as there was no effect of cue size in the tone absent condition (if anything it was in the opposite direction). Moreover, we found evidence that our attentional window manipulation was effective, since we observed a reliable effect of eccentricity that depended on the size of the attentional window. In other words, participants were better to find the synchronized target in the periphery when they adopted a large attentional window than when they adopted a small attentional window.

Whereas the size of an attentional window affected overall search times for synchronized audiovisual events, search slopes remained unaffected, and never reached values typically assumed for parallel search (for instance <10 ms/item [27]). Thus, even when participants distributed their attention, capture by audiovisual events was not perfect. This was also found by Belopolsky et al. [14], and explanations range from the inability to maintain a wide attentional distribution to a reluctance to adopt one in the first place. Note that without the tone, search is effortful and serial. In other words, the default setting to find the target is presumably a focused attention setting because without the sound increasing the salience of the target, the target is very hard to find. In the more classic singleton capture paradigm [12], [20], the target is always the unique pop-out element in the display, allowing participants to consistently adopt a diffuse attention window setting. In this classic task, one will obtain a completely flat search function. Also, in our previous study we have shown that the pip and pop effect is susceptible to whether participants made eye-movements, explaining the presence of some residual slopes as well ([6] Experiment 4). Regardless of the presence of residual search slopes, the present study clearly shows that the size of the attentional window modulates capture by audiovisual synchronization.

The present findings are consistent with the visual search literature. For instance, visual attention is readily drawn to visual objects that stand out from the background, such as a unique red object in a field of green objects, or an abrupt onset (e.g. [12], [13]). Even though many studies have shown that such salient features capture attention in an automatic, exogenous manner, recent evidence suggests that the size of the attentional window may modulate the extent to which salient events capture attention [14], [20], [21]. For instance, Theeuwes [20] showed that abrupt onsets do not capture attention when participants focus their attention on valid target location before display onset. Theeuwes suggested that when attention is in an unfocused, distributed state, attention covers the entire visual field. In contrast, an endogenous cue enables attention to “zoom in” on a particular area, which explains why abrupt onsets do not necessarily capture attention when such onsets are located outside the attentional area. Consistent with Theeuwes, we suggest that audiovisual events captures attention in an automatic manner, however, this capture depends on whether the visual event occurs within the attended area. This also explains why we observed an eccentricity effect of the pip and pop phenomenon, because on some trials the attentional window is not wide enough to enable capture by audiovisual synchrony.

Whereas many studies reported that multisensory integration occurs automatically [11], [28]–[30], and that multisensory integration can even guide attention [7], [10], [31], other studies have claimed quit the opposite, that some attention is beneficial (and sometimes even necessary) to establish binding from different modalities [32]–[38]. In a recent review, Talsma, Senkowski, Soto-Faraco and Woldorff [39] discussed the role of attention in multisensory processing. With regard to the pip and pop effect, they proposed that (p. 400): *“Stimulus-driven, bottom-up mechanisms induced by crossmodal interactions can automatically capture attention towards multisensory events, particularly when competition to focus elsewhere is relatively low.”* Consistent with this notion, we observed an effect of automatically driven capture as indexed by the pip and pop effect, and that this is stronger when participants adopt a large (i.e. less focussed) attentional window than when they are forced to focus to the small cue prior the first audiovisual event.

All in all then, the present study should temper our previous claims that the pip and pop effect occurs automatically independent of any top-down control [6], [9], [10]. The current findings suggest that the pip and pop effect occurs in an automatic fashion as long as spatial attention is divided across the visual field. The extent to which attention is divided across the visual field is under top-down control (Theeuwes, 1994, 2010). Our claims are consistent with the results of a study by Ngo and Spence [8] who replicated the pip and pop effect, and reported additional top-down cueing effects depending on the location of the synchronized auditory signal. So, in other words, search was even more improved when the location of the tone predicted the target location correctly than when the location of the tone predicted the target location incorrectly. Whereas Ngo and Spence reported spatial cueing effects (see also [40], [41]) when they manipulated the location of the synchronized auditory signal, we reported cueing effects when we manipulated the size of the visual cue prior to the spatially uninformative auditory signal. In the present study, the cue was always centrally presented and did not contain any spatial information about the target location.

The present findings may appear inconsistent with those of Santangelo and Spence [31]. They investigated whether unimodal and multimodal cues still capture attention when participants had to monitor a rapidly presented central stream of visual letters for occasionally presented digits. Under this high perceptual load condition, spatial cueing effects were observed when the visual target was preceded by a multimodal cue, but not when the visual target was preceded by a unimodal cue. The presence of a multisensory integration under focused attention conditions appears at odds with the present argument. However, we do not claim that under focused attention conditions there is no attentional capture by audiovisual events whatsoever. After all, observers were still better in the tone present condition than in the tone absent condition even under focused settings. All we claim is that capture is *reduced* under focused conditions relative to distributed attentional settings.

## References

[1] Alais D, Newell FN, Mamassian P (2010). Multisensory processing in review: from physiology to behaviour.. Seeing and perceiving.

[2] Shams L, Kim R (2010). Crossmodal influences on visual perception.. Physics of Life Reviews.

[3] Alais D, Burr D (2004). The ventriloquism effect results from near-optimal bimodal integration.. Current Biology.

[4] McGurk H, MacDonald J (1976). Hearing lips and seeing voices.. Nature.

[5] Spence C (2007). Audiovisual multisensory integration.. Acoustical Science and Technology.

[6] Van der Burg E, Olivers CNL, Bronkhorst AW, Theeuwes J (2008). Pip and pop: Non-spatial auditory signals improve spatial visual search.. Journal of Experimental Psychology: Human Perception and Performance.

[7] Van der Burg E, Olivers CNL, Bronkhorst AW, Theeuwes J (2009). Poke and pop: Tactile-visual synchrony increases visual saliency.. Neuroscience Letters.

[8] Ngo MK, Spence C (2010). Auditory, tactile, and multisensory cues facilitate search for dynamic visual stimuli.. Attention, Perception & Psychophysics.

[9] Van der Burg E, Talsma D, Olivers CNL, Hickey C, Theeuwes J (2011). Early multisensory interactions affect the competition among multiple visual objects.. NeuroImage.

[10] Van der Burg E, Olivers CNL, Bronkhorst AW, Theeuwes J (2008). Audiovisual events capture attention: Evidence from temporal order judgments.. Journal of Vision 8: art.

[11] Matusz PJ, Eimer M (2011). Multisensory enhancement of attentional capture in visual search.. Psychonomic Bulletin & Review.

[12] Theeuwes J (1992). Perceptual selectivity for color and form.. Perception & Psychophysics.

[13] Yantis S, Jonides J (1984). Abrupt visual onsets and selective attention: Evidence from visual search.. Journal of Experimental Psychology: Human Perception and Performance.

[14] Belopolsky AV, Zwaan L, Theeuwes J, Kramer AF (2007). The size of an attentional window modulates attentional capture by color singletons.. Psychonomic Bulletin & Review.

[15] Belopolsky AV, Theeuwes J (2010). No capture outside the attentional window.. Vision Research.

[16] Hernandez M, Costa A, Humphreys GW (2010). The size of an attentional window affects working memory guidance.. Attention, Perception & Psychophysics.

[17] Theeuwes J (1994). Stimulus-driven capture and attentional set: Selective search for color and visual abrupt onsets.. Journal of Experimental Psychology: Human Perception and Performance.

[18] Theeuwes J (1994). Endogenous and exogenous control of visual selection.. Perception.

[19] Theeuwes J (2010). Top-down and bottom-up control of visual selection.. Acta Psychologica.

[20] Theeuwes J (1991). Exogenous and Endogenous Control of Attention: The Effect of Visual Onsets and Offsets.. Perception & Psychophysics.

[21] Yantis S, Jonides J (1990). Abrupt visual onsets and selective attention: Voluntary versus automatic allocation.. Journal of Experimental Psychology: Human Perception and Performance.

[22] Yantis S, Johnston JC (1990). On the Locus of Visual Selection: Evidence From Focused Attention Tasks.. Journal of Experimental Psychology: Human Perception and Performance.

[23] Carrasco M, Evert DL, Chang I, Katz SM (1995). The eccentricity effect: Target eccentricity affects performance on conjuction searches.. Perception & Psychophysics.

[24] Van der Burg E, Cass J, Olivers CNL, Theeuwes J, Alais D (2010). Efficient visual search from synchronized auditory signals requires transient audiovisual events.. PLoS ONE.

[25] Staufenbiel SM, van der Lubbe RHJ, Talsma D (2011). Spatially uninformative sounds increase sensitivity for visual motion change.. Experimental Brain Research.

[26] de Boer-Schellekens L, Vroomen J (2011). Sound can improve visual search in developmental dyslexia.. Experimental Brain Research.

[27] Treisman A, Gelade G (1980). A feature-integration theory of attention.. Cognitive Psychology.

[28] Driver J (1996). Enhancement of selective listening by illusory mislocation of speech sounds due to lip-reading.. Nature.

[29] Vroomen J, De Gelder B (2000). Sound enhances visual perception: Cross-modal effects of auditory organization on vision.. Journal of Experimental Psychology: Human Perception and Performance.

[30] Olivers CNL, Van der Burg E (2008). Bleeping you out of the blink: Sound saves vision from oblivion.. Brain Research.

[31] Santangelo V, Spence C (2007). Multisensory cues capture spatial attention regardless of perceptual load.. Journal of Experimental Psychology: Human Perception and Performance.

[32] Alsius A, Navarra J, Soto-Faraco S (2007). Attention to touch weakens audiovisual speech integration.. Experimental Brain Research.

[33] Alsius A, Navarra J, Campbell R, Soto-Faraco S (2005). Audiovisual integration of speech falters under attention demands.. Current Biology.

[34] Busse L, Roberts KC, Crist RE, Weissman DH, Woldorff MG (2005). The spread of attention across modalities and space in a multisensory object.. Proceedings of the National Academy of Science.

[35] Fujisaki W, Koene A, Arnold D, Johnston A, Nishida S (2006). Visual search for a target changing in synchrony with an auditory signal.. Proceedings of the Royal Society B: Biological Sciences.

[36] Talsma D, Doty TJ, Woldorff MG (2007). Selective attention and audiovisual integration: Is attending to both modalities a prerequisite for early integration?. Cerebral Cortex.

[37] Van Ee R, Van Boxtel JJA, Parker AM, Alais D (2009). Multisensory Congruency as a Mechanism for Attentional Control over Perceptual Selection.. The Journal of Neuroscience.

[38] Van der Burg E, Brederoo SG, Nieuwenstein MR, Theeuwes J, Olivers CNL (2010). Audiovisual semantic interference and attention: Evidence from the attentional blink paradigm.. Acta Psychologica.

[39] Talsma D, Senkowski D, Soto-Faraco S, Woldorff MG (2010). The multifaceted interplay between attention and multisensory integration.. Trends in Cognitive Sciences.

[40] Perrott DR, Cisneros J, McKinley RL, D’Angelo WR (1996). Aurally aided visual search under virtual and free-field listening conditions.. Human Factors.

[41] Spence C, Driver J (1996). Audiovisual links in endogenous covert spatial attention.. Journal of Experimental Pscyhology: Human Perception and Performance.

